# Community Involvement in Dengue Outbreak Control: An Integrated Rigorous Intervention Strategy

**DOI:** 10.1371/journal.pntd.0004919

**Published:** 2016-08-22

**Authors:** Hualiang Lin, Tao Liu, Tie Song, Lifeng Lin, Jianpeng Xiao, Jinyan Lin, Jianfeng He, Haojie Zhong, Wenbiao Hu, Aiping Deng, Zhiqiang Peng, Wenjun Ma, Yonghui Zhang

**Affiliations:** 1 Guangdong Provincial Institute of Public Health, Guangdong Provincial Center for Disease Control and Prevention, Guangzhou, China; 2 Guangdong Provincial Center for Disease Control and Prevention, Guangzhou, China; 3 School of Public Health and Social Work, Queensland University of Technology, Australia; 4 Institute of Health and Biomedical Innovation, Queensland University of Technology, Australia; University of California, Berkeley, UNITED STATES

## Abstract

**Background:**

An explosive outbreak of dengue fever occurred in Guangdong Province, China in 2014. A community-based integrated intervention was applied to control this outbreak in the capital city Guangzhou, where dengue epidemic was mainly caused by imported cases.

**Methodology/Principal Findings:**

We used a time series generalized additive model based on meteorological factors to assess the effectiveness of this intervention. The results showed that there was significant reduction in mosquito density following the intervention, and there was a 70.47% (95% confidence interval: 66.07%, 74.88%) reduction in the reported dengue cases compared with the predicted cases after 12 days since the beginning of the intervention, we estimated that a total of 23,302 dengue cases were prevented.

**Conclusions:**

This study suggests that an integrated dengue intervention program has significant effects to control a dengue outbreak in areas where dengue epidemic was mainly caused by imported dengue cases.

## Author Summary

Dengue fever, caused by any of the four dengue virus serotypes, is regarded as one of the most important arboviral diseases globally. Guangdong Province in south China has the highest dengue infestation level in mainland China. An explosive outbreak occurred in this province in 2014, with a total of 36,837 cases and 6 deaths being notified. A community-based integrated intervention program was implemented to control this outbreak in Guangzhou, the capital city of the province, where dengue epidemic was mainly caused by imported cases. It was estimated that a total of 3.3 million people and 272 million RMB were invested in this intervention. This study used a time series generalized additive model based on meteorological factors to evaluate the effectiveness of this intervention program. The analysis showed that there was significant reduction in mosquito density following the intervention (Breteau Index (BI) reduced from 10.88 to 2.11), and there was a 70.47% (95% confidence interval: 66.07%, 74.88%) reduction in the reported dengue cases compared with the predicted cases after 12 days since the beginning of the intervention. A total of 23,302 dengue cases were prevented due to the community-based intervention. This study suggests that an integrated dengue intervention program is effective to control a dengue outbreak in areas where dengue epidemic was mainly caused by imported dengue cases.

## Introduction

Dengue fever, a mosquito-borne viral disease caused by any of the four dengue virus serotypes, is regarded as one of the most important arboviral diseases globally [1]. At present, no specific antiviral treatment or vaccine against dengue fever is available. It was estimated that about 2,500 million people live in areas at the threat of dengue infection worldwide [2]. Currently, Dengue fever distributes in most tropical countries of the South Pacific, Asia, the Caribbean, the Americas, and Africa; the importance of dengue to public health is growing rapidly due to its geographical expansion probably resulting from population growing, increasing population movement, environmental change, particularly climate change [3,4].

Guangdong Province has the highest dengue infestation level in mainland China [5]. In 1978, dengue fever re-emerged in Guangdong Province after disappearing for about 30 years in mainland China. Since then, it occurred in Guangdong almost every year [6,7]. The dengue cases in this province were mainly caused by imported cases from surrounding dengue endemic countries and areas [8].

An unprecedented explosive outbreak of dengue fever occurred in Guangdong Province in 2014; the case number was more than 10 times of the total number in previous 10 years with 6 deaths [9]. During the initial stage before September 23, only routine control measures were implemented around the outbreak sites, which focused on insecticide space-spraying. However, since September 23, the increasing intensity and spatial expansion prompted the government to take a comprehensive integrated community-based control strategy [7]. A comparison of the two strategies was illustrated in S1 Table. Briefly, compared with the routine control measures, the integrated community-based control strategy is to mobilize all community partners to participate in the dengue control activity with the highest administrative leadership and support, and to monitor the mosquito density in all public places and to control the density at a safe level.

Evaluation of the effectiveness of the intervention measures, referred to as accountability research, has been increasingly viewed as a necessary component of responsible governmental policy [10]. This study aims to assess the effectiveness of the comprehensive and intensified control measures based on community in Guangzhou, the capital city of Guangdong Province, China.

## Materials and Methods

### Study setting

Guangzhou, the capital city of Guangdong Province, is situated in the southern China. It has an area of 7434 km^2^ and about 12.7 million inhabitants. The climate is subtropical humid, with an average annual temperature of 21.9°C, the highest mean temperature (33.0–34.9°C) is observed between July and August and the lowest mean temperature (6.5–12.1°C) between January and February, the annual average rainfall of 1500 to 2000 mm. This city has short, mild, dry winters and long, hot, wet summers.

### Ethical review

The present study was reviewed and approved by the Medical Ethics Committee of Guangdong Provincial Centre for Disease Control and Prevention. All the participants' medical data were anonymized, as we only used the daily number of dengue fever cases for this study.

### Integrated intervention strategy

Integrated community-based control strategy required every community to be involved in the dengue fever control activity under the leadership of the government; a multi-sectoral collaboration mechanism was established; the health authority was responsible for the technical organization and inspection. Here, a community is defined as a residential unit situated in a given geographical area with an administrative organization, the geographic size may vary greatly. The integrated intervention measures included larval breeding eradication, killing adult mosquitoes with pesticides, public health education and community involvement, as well as rigorous administrative leadership. Taking Guangzhou as an example, a set of special financial support and resources was allocated for this intervention, it was estimated that a total of 3.3 million people have participated in this activity and 272 million RMB were spent to purchase pesticides and related instruments. This rigorous strategy was organized by the provincial and municipal governments, which convened the Special Dengue Control Committee for oversight, with technical advice and training. The Committee team and relevant provincial and municipal professionals met with health department staff to propose and discuss the strategy and to gain initial consent. After approval had been given, the horizontal component was implemented by the health personnel of each community, park, school teachers, etc; community members were also mobilized to do household cleaning, particularly water container management. The district and community health officials were responsible for coordination of different sectors, inspection, evaluation, summary of the field activity, and delivery of health education messages to the public. Each community was obliged to establish a dengue control team to implement standard dengue control activities: entomological surveillance and breeding source reduction through periodic inspection of houses, larviciding of various containers, adult mosquito density control, communication and education on dengue prevention, and enforcement of mosquito control legislation.

All the public places, including hospitals, schools, parks, public squares and tourist sites, were requested to do mosquito density survey and report the survey results to the local health department every day. The school teachers and students were also required to participate in the clean-up campaigns, the children were educated to provide knowledge and control approaches to their family members, and participation in dengue or project-oriented plays, songs, quiz, and so on.

### Data collection

Dengue fever has been a legally notifiable communicable disease in China since 1989. Daily records of dengue cases between 2006 and 2014 in Guangzhou were obtained from the China National Notifiable Disease Reporting System. The information included age, sex, occupation, date of symptom onset, whether the diagnosis was clinical or confirmed by laboratory test, etc.

In the study area, *Aedes albopictus* is the dominant transmission vector [11]. Herein, Breteau Index (BI), one of the accepted indicators for Aedes density [12], was collected from various districts in Guangzhou. From each district, three streets were selected as the BI monitoring points and water containers and mosquito larvae were checked from 100 houses through a weekly survey; the houses for the survey remained consistent during the study period. BI is calculated according to the number of positive containers per 100 houses inspected.

Daily meteorological data, including mean temperature, relative humidity from 2006 to 2014, were retrieved from the China Meteorological Data Sharing Service System (http://cdc.cma.gov.cn/index.jsp).

### Statistical analysis

To evaluate the effectiveness of this integrated intervention program, we collected daily data on dengue fever and meteorological variables (daily mean temperature (°C) and relative humidity (%)) in Guangzhou for the period January 1, 2006 through December 31, 2014. A generalized additive model with a quasi-Poisson link function to account for over-dispersion in daily dengue cases was utilized to establish the predicting model. In the model, the daily number of dengue cases was treated as dependent variable, and daily meteorological variables with certain lag days, temporal trend and public holidays (PH) were used as explanatory variables. Public holidays was defined as the holidays and weekend when people don't need to work. To control for the non-linear relationship between the explanatory variables and dengue fever, we used a smoothing function based on penalized splines for temporal trend and meteorological factors [13], the degrees of freedom of the smoothing function were selected based on previous studies [14]. For example, we initially applied 7 df per year for time trends to filter out the information at time scales of longer than two months, 6 df for mean temperature, and 3 df for relative humidity. The model can be specified as:

$$\text{log}\left[E(Y_{t})\right]\;=\alpha +\beta_{1}*\text{AR}\left(\text{dengue},\;1\right)\;+\text{ s}\left(\text{t},\text{ df}=7/\text{year}\right)\;+\text{ s}\left({\text{Temp}}_{\text{n}},\text{ df }=6\right)\;+\text{ s}\left({\text{Humidity}}_{\text{n}},\text{ df}=3\right)\;+\beta_{2}*\text{PH}$$

where E(Y_t_) is the expected number of dengue cases on day t, AR(dengue, 1) is the term of auto-regression of dengue cases of previous day, α is the model intercept, s() indicates a smoother based on penalized splines, df is the degree of freedom, t represents time to adjust for long-term trend and seasonality, Temp_n_ is the mean temperature on a lag of n days, Humidity_n_ presents the relative humidity on a lag of n days, the lag days ranged from 14 to 30 days according to the transmission pattern of this disease [15]; PH represents a binary variable for the public holidays, β is the regression coefficient. The model specification in terms of lag days of meteorological variables and degree of freedom for smoothing function was determined using the R square (R^2^) criteria, the higher of the R^2^ value, the better model fit.

We established the model using the data from January 1, 2006 through September 23, 2014, which was then used to predict the dengue epidemic during August 25 to 31 December, 2014. The reduction rate was calculated by comparing the predicted dengue cases with the actual cases.

The sensitivity of the effect estimates was assessed in terms of the degrees of freedom of the smoothing function of temporal trends (5, 6 and 8 per year) and meteorological variables, including mean temperature (df = 4, 5 and 7) and relative humidity (df = 4–6). We also fitted the model using observation data of different cut-off time points (for example, from January 1, 2006 through September 5, 2014, and through September 15, 2014). We also applied an SEIR (Susceptible, Exposed, Infected and Removed) model to do the analysis to check the robustness of the effect estimate, the details of the method were shown in the supplementary materials.

## Results

During the study period, a total of 39,214 dengue cases were reported in Guangzhou. Among them, 36,837 were notified in 2014 with an incidence of 290 per 100,000 population, accounting for 93.94% of the total cases during the study period. There were slightly more female cases with male-to-female sex ratio of 0.96:1 (19248:19966).

**Fig 1:**
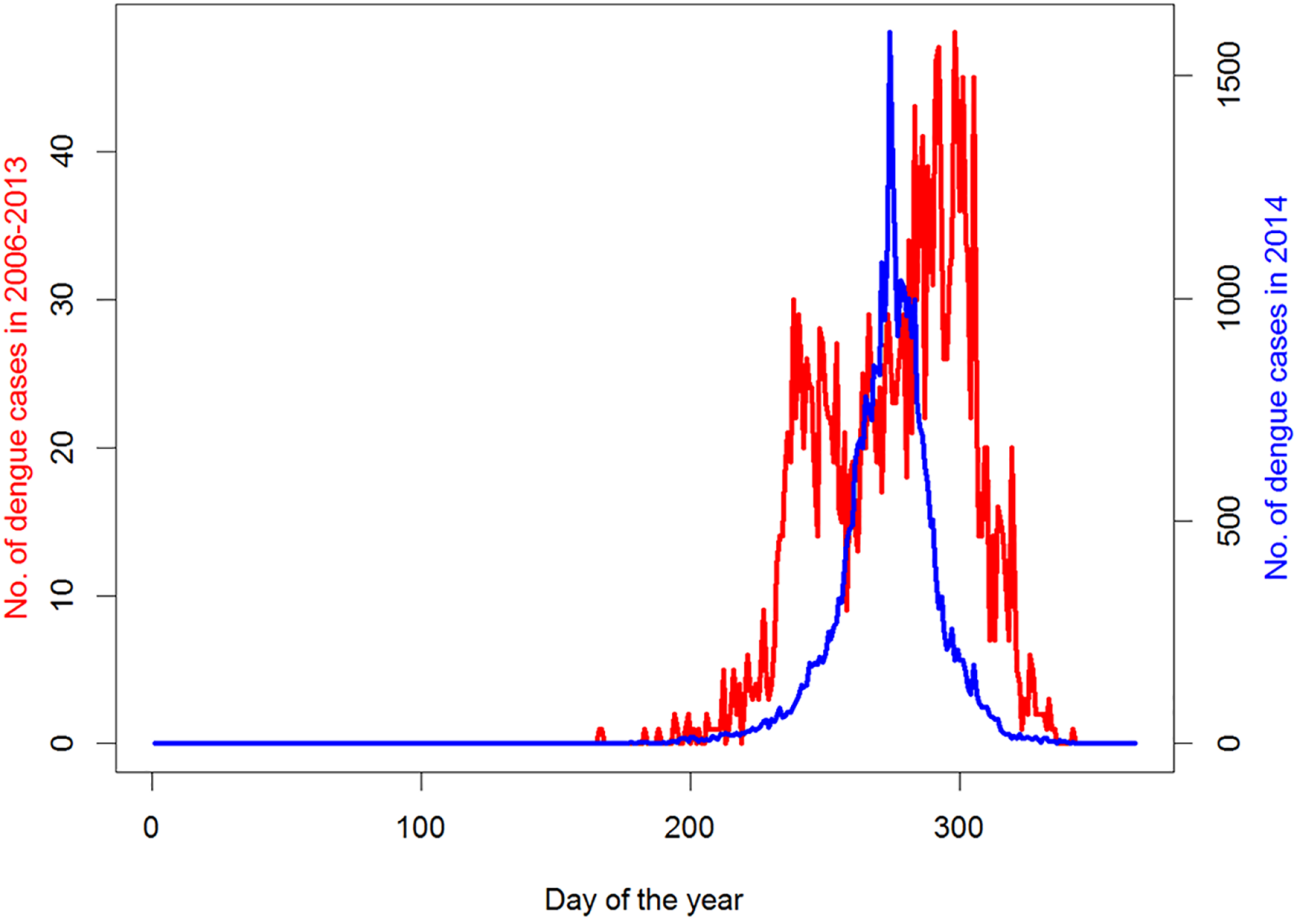
Comparison of the time series of dengue fever in 2006–2013 and 2014 in Guangzhou.

From 2006 to 2014, there were three epidemic years in which the number of annual dengue cases reached more than 700 (i.e., 774 cases in 2006, 1,268 cases in 2013, and 36,837 cases in 2014). The incidence of dengue fever presented an obvious seasonal pattern with higher rate occurring from June to November (Fig 1).

**Fig 2:**
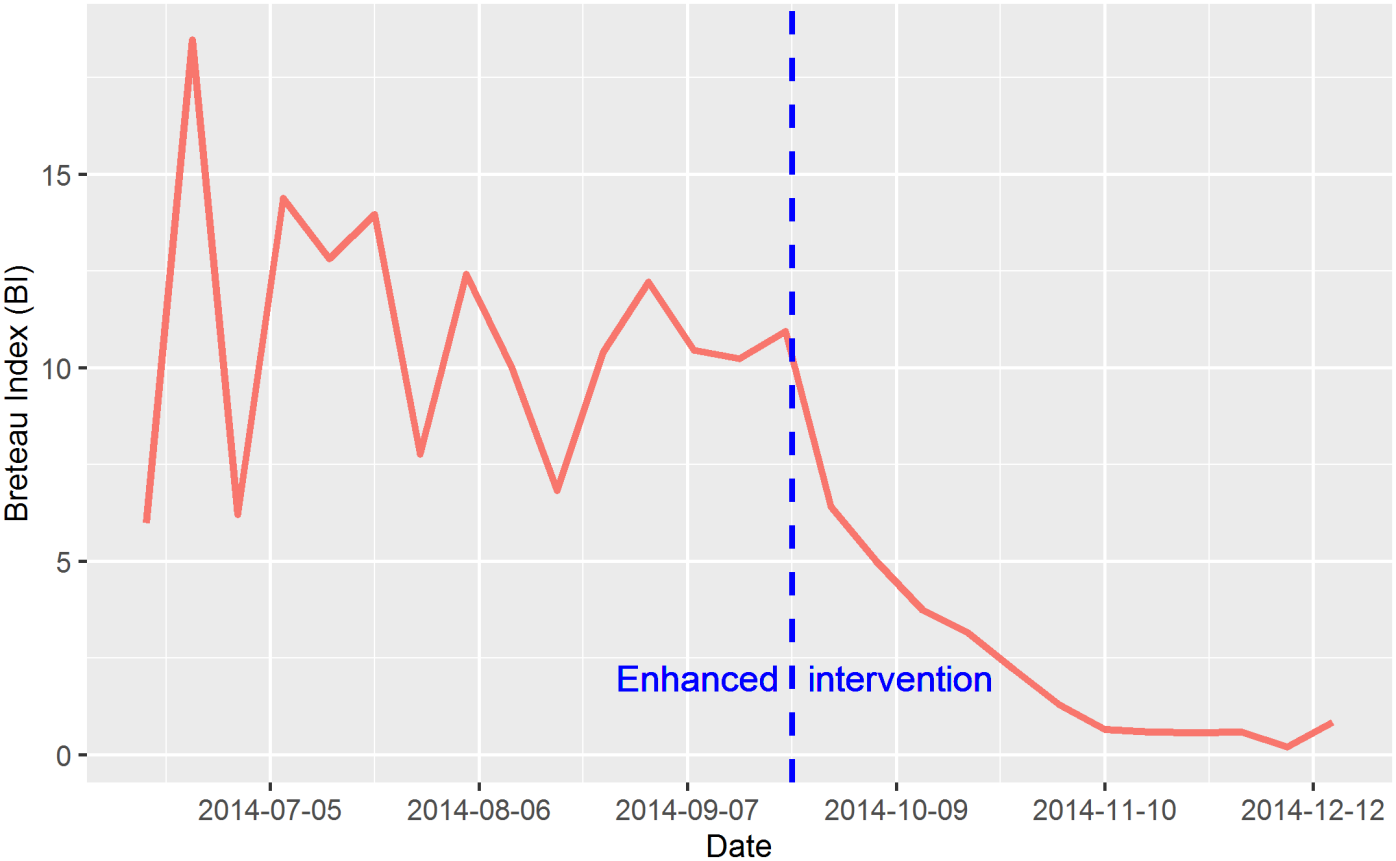
Temporal trend of in Breteau Index during the period of June 2014 through December 2014 in Guangzhou.

Fig 2 shows the temporal trend of Breteau Index during the period of June 2014 through December 2014. The average index was 10.88 before the integrated intervention, after which, the index decreased to an average of 2.11. An exponential decay model suggested that the Breteau index decreased in an exponential function (alpha = 633.332, gamma = -0.039) after the intervention.

The model with moving average of 7–28 lag days’ meteorological variables and df of 7 for temporal trend, 6 for temperature and 3 for relative humidity was found to have the best model fit with R^2^ being 99.4% (internal validity).

**Fig 3:**
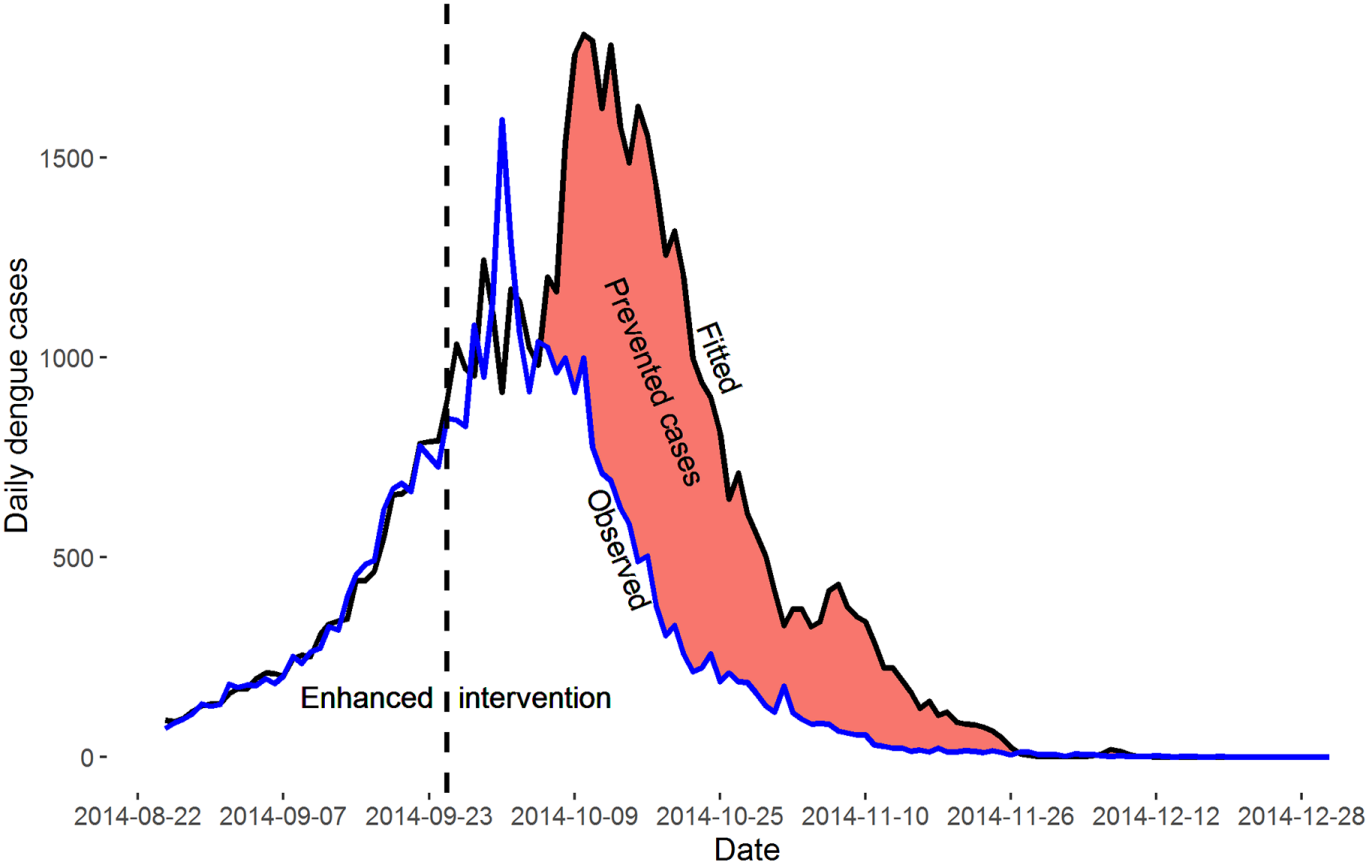
The observed and predicted dengue fever cases in Guangzhou, 2014.

During the outbreak in 2014, the epidemic peak was observed on October 1 with 1,596 dengue cases, after that the epidemic began to decrease; while our predicted epidemic peak was estimated on October 5 with 1,810 dengue cases (Fig 3). The comparison of the epidemic curves in 2006–2013 and 2014 also supported that the epidemic peak in 2014 was relatively earlier than that in 2006–2013.

The reduction rate of daily dengue cases for October 5 to November 26 was 70.47% (95% confidence interval (CI): 66.06%, 74.88%), about 23,302 dengue cases have been prevented. When we used alternative time period to examine the effect estimates, the reduction rate was 61.10% (95% CI: 57.26%, 70.95%), and about 22,641 dengue cases might have been prevented for the period of October 1 to December 31, and 75.71% (95% CI: 73.26%, 78.17%), and the prevented dengue cases were 21,539.

Sensitivity analyses found that the effect estimates were relatively robust to the degrees of freedom of smoothing adjustment for temporal trend and weather variables (S2 Table). For example, when we used 6 of df for temporal adjustment, the reduction rate was 70.43% (95% CI: 66.02%, 74.84%) and an estimated 23,244 dengue cases might have been prevented by the intervention. And when using data of different time periods to fit the model, we also obtained similar effect estimates, for example, when we used data from January 1, 2006 through September 15, 2014 to construct the model, the reduction rate was 70.18% (95% CI: 65.67%, 74.70%), and the prevented dengue cases were 22,348. The SEIR model also yielded a consistent result with that of main model (as shown in S1 Fig), which estimated that 25,532 dengue cases might have been prevented.

## Discussion

For the past few decades, vector control methods to reduce mosquito breeding sites and density remained the mainstay of prevention and control of dengue fever [16]. This approach, however, is usually of questionable efficacy and is often inefficient due to absence of active community involvement [17]. Alternative approaches emerged in recent years, including genetically-modified mosquitoes, biological control methods (such as Wolbachia), anti-viral drugs and vaccines [18]. The present study indicated that the comprehensive and intensified dengue intervention strategy based on community participation was effective to rapidly reduce the mosquito density and curtail the dengue outbreak in an area where dengue epidemic was mainly caused by imported cases.

Routine dengue control measures rely mainly on vector control and generally consists of source reduction, larviciding and/or insecticide space-spraying [19]. However, the vector control strategy usually lacks effectiveness and sustainability [20], while community involvement and enhanced government leadership strategies have been proved to be successful in a few countries, which included systematic human resources and prevention facility investment and scientific allocation of these resources [21]. Historically, there have been only a few examples of successful dengue prevention through vector control [22–24]. The first one was the highly successful, vertically structured paramilitary hemispheric eradication campaign directed by the Pan American Sanitary Board from 1946 to 1970 [22]. The second was also a rigorous, top-down, military-like vector control program in Cuba, which was based on intensive insecticidal treatment followed by larval habitat management in 1981 [23]. The third successful program was reported in Singapore [24]. Therefore, assessing some new dengue control strategies is very important to provide potentially high-impact interventions for resource-poor countries where dengue is a major public-health problem.

The unsuccessful vector control strategy in some countries might be due to that the vector control strategy was unsustainable or lack of sufficient community participation, but did not necessarily mean that vector control measures were unable to reduce transmission. Actually, the experiences from Vietnam have showed integrated vector control strategy based on community involvement was effective in prevention and control of dengue fever epidemic [25].

Although the integrated intervention strategy has proven to be successful in Guangzhou, one challenge is how to keep the program sustainable by activating each component of the integrated community-based strategy in the provincial dengue contingency plan when an outbreak is first suspected. The strategy should also be considered by other countries and areas with similar situation as Guangzhou, such as the dengue outbreak was caused by imported dengue cases, and similar hierarchical structure to adopt the strategy. The hierarchical structure and social-economic status in different countries could affect successful adoption of the strategy; the key components rely on motivating community and individual engagement, which highly depends on their perception of the severity of the disease, and willingness to take responsibility. Among them, barriers to sustaining dengue vector control actions are significant and include, among others, onerous behaviors that must be carried out on a weekly basis, and which may not be perceived as beneficial given the time needed to carry them out; other barrier included some breeding sources of mosquitoes in the community that are not amenable to individual control and thus serve as a reminder to the community that their neighborhood lacks good quality public services; ineffective vector control strategies due to budget and personnel limitations. Along with the increasing importance of dengue fever, the authors predict that the comprehensive dengue intervention model, or modifications of it, will become increasingly important.

In summary, this study suggests that a comprehensive and enhanced dengue intervention strategy based on community engagement has significant effect to control a dengue outbreak in areas where the dengue epidemic was mainly caused by imported cases.

## Supporting Information

S1 Fig The observed and predicted dengue fever cases estimated by an SEIR model in Guangzhou, 2014. (DOC)

S1 Table Comparison of the key components of the routine dengue control measures and the integrated community-based control strategy in Guangzhou, China. (DOC)

S2 Table Effect estimation for the effectiveness of the intervention using different model specifications. (DOC)
