# Supplementary material for: Community Involvement in Dengue Outbreak Control: An Integrated Rigorous Intervention Strategy
Source: PLoS Negl Trop Dis. 2016 Aug 22;10(8):e0004919. doi: 10.1371/journal.pntd.0004919 (PMC4993447; doi:10.1371/journal.pntd.0004919)
Supplement: S1 Fig — (DOC) [file pntd.0004919.s001.doc]

We used a SEIR (Susceptible, Exposed, Infected and Removed) model to simulate the daily dengue cases under scenarios of routine and enhanced control strategies, and further assessed the effectiveness of the enhanced community-involvement strategy. The SEIR model is a compartmental dynamic model that simulates daily dengue cases under specific conditions. This model divides the total human population (N_h_) into four groups [[9](#_ENREF_9)]: people who are susceptible to the infection with dengue virus (S_h_), people who have been exposed to the virus (E_h_), people who are infected (I_h_) and people who have recovered (R_h_). Adult female mosquitoes (N_v_) can also be categorized as those that are susceptible with dengue virus (S_v_), mosquitoes that have been exposed to the virus (E_v_), and mosquitoes that are infected with dengue virus (I_v_). Humans enter the susceptible class through birth, and become infected with a finite probability after being bitten by an infectious mosquito. After being successfully infected, humans move from the susceptible group (S_h_) to the exposed group (E_h_), and after an intrinsic incubation period, they move to the infectious class (I_h_). Finally after a period of time, these humans recover and move to the recovered class (R_h_).

In this study, we used the daily Breteau Index (BI) as an indicator to reflect the intensity of control measures. The BI data were divided into two periods: routine control period before September 25, 2014, and enhanced control period after September 25, 2014. The BIs were 10.80 and 1.86 during the two periods, respectively. Therefore, the ratio (1.86/10.80*100=17.2%) was used as a parameter in the SEIR model to evaluate change of dengue cases between the routine and rigorous control interventions. The results indicated that if the routine control measures were implemented throughout the whole period, the number of cases would be 54,438. However, if the control strategy was intensified after September 25^th^, the total number of dengue cases would be 28,906 (as shown in Figure s1). Therefore, 25,532 (54,438-28,906) dengue cases were estimated to have been prevented.


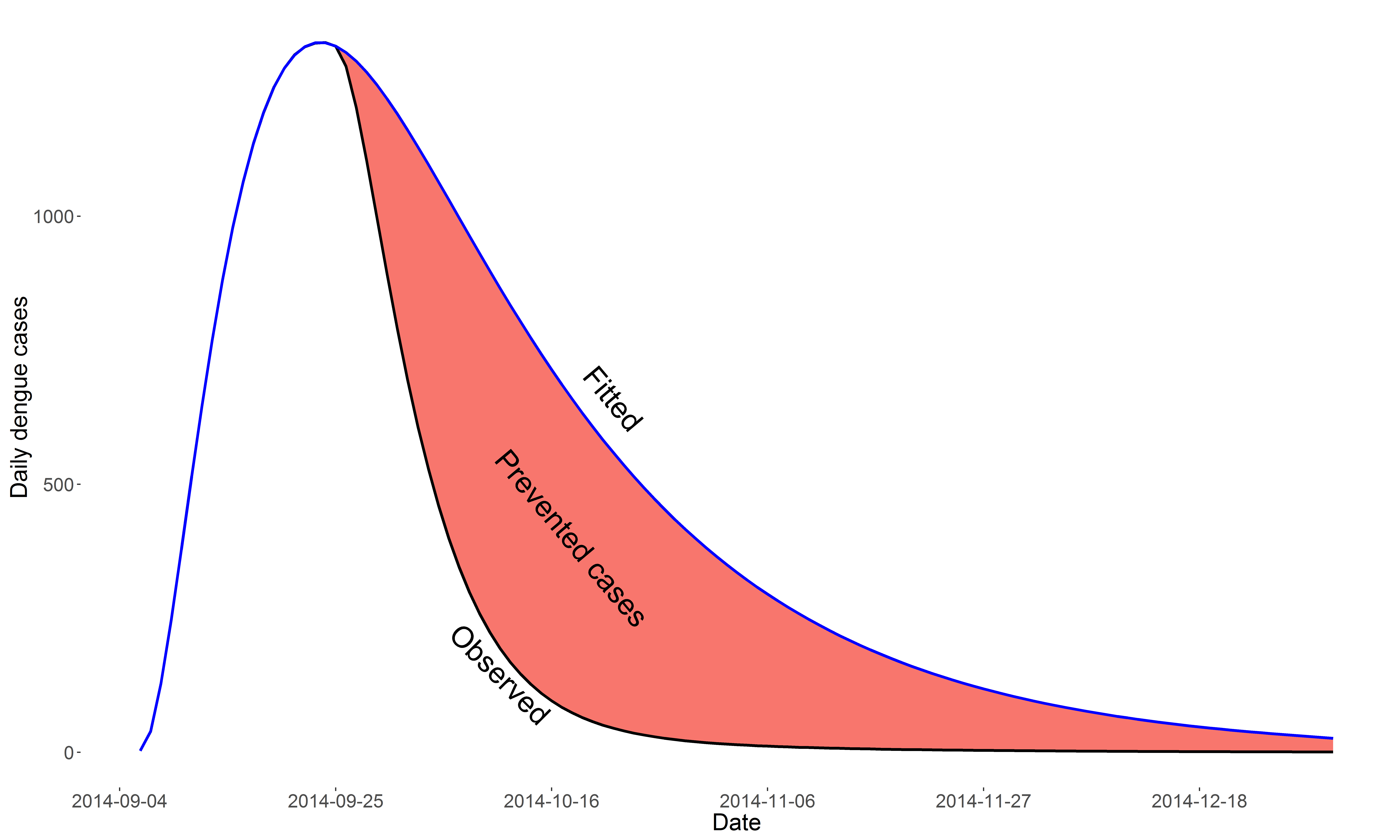


**Figure s1**. The observed and predicted dengue fever cases estimated by an SIR model in Guangzhou, 2014.
