# Supplementary material for: Community Involvement in Dengue Outbreak Control: An Integrated Rigorous Intervention Strategy
Source: PLoS Negl Trop Dis. 2016 Aug 22;10(8):e0004919. doi: 10.1371/journal.pntd.0004919 (PMC4993447; doi:10.1371/journal.pntd.0004919)
Supplement: S1 Table — (DOC) [file pntd.0004919.s002.doc]

Table s1. Comparison of the key components of the routine dengue control measures and the integrated community-based control strategy in Guangzhou, China

| **Key items** | **Routine control measures** | **Integrated community-based control strategy** |
| --- | --- | --- |
| Sectors involved | Mainly public health sectors | All community sectors |
| Participants | Mainly health professionals | All community members |
| Administrative leadership | Health authority | The government head |
| Administrative support | General | Highest |
| Multi-sectoral collaboration | Without | Established |
| Vector control team | Organized by health department | Organized by government with qualified techniques and facilities |
| Funding support | General | Highest priority |
| Mosquito density monitor | Only in a few fixed sites | In all public places |
| Mosquito control action | Only around the outbreak sites | In all potential outbreak areas |
