# Supplementary material for: Community Involvement in Dengue Outbreak Control: An Integrated Rigorous Intervention Strategy
Source: PLoS Negl Trop Dis. 2016 Aug 22;10(8):e0004919. doi: 10.1371/journal.pntd.0004919 (PMC4993447; doi:10.1371/journal.pntd.0004919)
Supplement: S2 Table — (DOC) [file pntd.0004919.s003.doc]

**Table s2. Effect estimation for the effectiveness of the intervention using different model specifications**

| Model | Reduction rate (%, 95% CI) | Prevented cases |
| --- | --- | --- |
| df for temporal trend |  |  |
| df=5 | 74.72 (70.77, 78.66) | 32484 |
| df=6 | 70.43 (66.02, 74.84) | 23244 |
| df=8 | 64.01 (59.31, 68.72) | 19630 |
| df for temperature |  |  |
| df=5 | 70.50 (66.09, 74.91) | 23333 |
| df=7 | 70.86 (66.41, 75.30) | 23400 |
| df=8 | 74.10 (69.54, 78.66) | 27061 |
| df for relative humidity |  |  |
| df=4 | 70.51 (66.15, 74.88) | 23294 |
| df=5 | 70.93 (66.46, 75.40) | 23607 |
| df=6 | 72.46 (68.26, 77.02) | 26841 |
| Model fitting data period |  |  |
| 1/1/2006-9/5/2014 | 71.01 (66.21, 75.81) | 22224 |
| 1/1/2006-9/15/2014 | 70.18 (65.67, 74.70) | 22348 |
